# Importance of Bcl-2-family proteins in murine hematopoietic progenitor and early B cells

**DOI:** 10.1038/s41419-021-04079-8

**Published:** 2021-08-11

**Authors:** Constanze Kurschat, Arlena Metz, Susanne Kirschnek, Georg Häcker

**Affiliations:** 1 Faculty of Medicine, Institute of Medical Microbiology and Hygiene, Medical Center, University of Freiburg, Freiburg, Germany; 2 BIOSS Centre for Biological Signalling Studies, University of Freiburg, Freiburg, Germany

**Keywords:** Apoptosis, Immune cell death

## Abstract

Mitochondrial apoptosis regulates survival and development of hematopoietic cells. Prominent roles of some Bcl-2-family members in this regulation have been established, for instance for pro-apoptotic Bim and anti-apoptotic Mcl-1. Additional, mostly smaller roles are known for other Bcl-2-members but it has been extremely difficult to obtain a comprehensive picture of the regulation of mitochondrial apoptosis in hematopoietic cells by Bcl-2-family proteins. We here use a system of mouse ‘conditionally immortalized’ lymphoid-primed hematopoietic progenitor (LMPP) cells that can be differentiated in vitro to pro-B cells, to analyze the importance of these proteins in cell survival. We established cells deficient in Bim, Noxa, Bim/Noxa, Bim/Puma, Bim/Bmf, Bax, Bak or Bax/Bak and use specific inhibitors of Bcl-2, Bcl-X_L_ and Mcl-1 to assess their importance. In progenitor (LMPP) cells, we found an important role of Noxa, alone and together with Bim. Cell death induced by inhibition of Bcl-2 and Bcl-X_L_ entirely depended on Bim and could be implemented by Bax and by Bak. Inhibition of Mcl-1 caused apoptosis that was independent of Bim but strongly depended on Noxa and was completely prevented by the absence of Bax; small amounts of anti-apoptotic proteins were co-immunoprecipitated with Bim. During differentiation to pro-B cells, substantial changes in the expression of Bcl-2-family proteins were seen, and Bcl-2, Bcl-X_L_ and Mcl-1 were all partially in complexes with Bim. In differentiated cells, Noxa appeared to have lost all importance while the loss of Bim and Puma provided protection. The results strongly suggest that the main role of Bim in these hematopoietic cells is the neutralization of Mcl-1, identify a number of likely molecular events during the maintenance of survival and the induction of apoptosis in mouse hematopoietic progenitor cells, and provide data on the regulation of expression and importance of these proteins during differentiation along the B cell lineage.

## Introduction

Mitochondrial apoptosis regulates many biological processes and is very important for differentiation and regulation of survival in hematopoietic cells [1]. Mitochondrial apoptosis is regulated by the Bcl-2-family, comprising three groups of proteins that can be distinguished by their structure and by their function. The pro-apoptotic group of Bax and Bak serve as the effectors, initiating the activation of effector caspases in the cytosol. The five anti-apoptotic proteins, Bcl-2, Bcl-X_L_, Bcl-w, Mcl-1 and A1, inhibit apoptosis by binding pro-apoptotic family proteins. The third group, known as BH3-only proteins and made up by eight members, are the initiators of apoptosis. BH3-only proteins trigger apoptosis through one of two mechanisms, by inhibiting anti-apoptotic Bcl-2-proteins and/or directly activating Bax/Bak. At least three BH3-only proteins, Bim, tBid (the active form of Bid) and Puma, can directly activate, while all BH3-only proteins (including Noxa, Bad, Bmf, Bik and Hrk) can inhibit anti-apoptotic Bcl-2-proteins [1, 2].

These basic operative principles of mitochondrial apoptosis seem unequivocal. It has however been notoriously difficult to move towards a detailed understanding of the events of the initiation of apoptosis and the chain of events from the receipt of a pro-apoptotic stimulus, over the activation of one or more BH3-only proteins and over the potentially necessary neutralization of anti-apoptotic Bcl-2 proteins to the activation of Bax or Bak or both. It is experimentally extremely challenging to control for the multitude of proteins and potential activation steps. Very likely, the apoptotic process is further regulated differently in different cell types. Gene-deletion experiments with mice and analysis of cell populations in physiological or near-physiological conditions have provided information on the roles of Bcl-2-family proteins, for instance by measuring cell differentiation and population size in vivo or by testing of apoptosis induced by lack of signaling input in vitro. Cell biological and biochemical studies mostly in tumor cell lines have yielded results in terms of interaction between Bcl-2-family members and sometimes activation studies. The relatively recent development of specific inhibitors of anti-apoptotic Bcl-2-members has facilitated the study of mitochondrial apoptosis.

Mitochondrial apoptosis is a major regulator of homeostasis of immune cell populations [3]. Loss of individual anti-apoptotic proteins causes cell death in some cell populations [4], and at least some Bcl-2-like proteins can compensate for each other [5]. In terms of BH3-only proteins, the loss of Bim has the most severe effect, leading to enhanced apoptosis resistance in several cell types [6, 7]. Some role has also been attributed to other BH3-only proteins, and loss of Puma [8], Bmf [9] or Noxa [10, 11] on a Bim-negative background has been found to have varying effects. The knowledge of the exact molecular function of Bcl-2-family proteins and how they neutralize or activate other family members to trigger apoptosis is far from comprehensive.

In this study, we endeavored to obtain information on the role of Bcl-2-family proteins in mouse lymphoid-primed hematopoietic progenitor cells (LMPP) and cells differentiating from these progenitors towards the B cell lineage. LMPP represent a stadium of hematopoietic differentiation, of committed progenitors with both myeloid and lymphoid potential [12]. We use a system of ‘conditionally immortalized’ mouse LMPP, which on one hand behave very similarly to primary cells [13] and which, on the other hand, permit the cell biological study of mitochondrial apoptosis. We use cells deficient in pro-apoptotic Bcl-2-family proteins or in Bax/Bak, and employ Bcl-2-protein inhibitors to test for the role of these components in apoptosis regulation and to probe some Bcl-2-protein family interactions. We follow the differentiation to early B cells in vitro, monitor protein-expression and their changing importance during differentiation.

## Results

### Contribution of BH3-only proteins to apoptotic signals in FLT3-progenitor lines

Hoxb8 is a homeobox family protein that can increase self-renewal and arrest differentiation in hematopoietic cells [14]. A Hoxb8-variant that is fused to the ligand binding domain of the estrogen-receptor can be turned on and off by adding or washing away estrogen [15]. When this construct is expressed in mouse hematopoietic progenitor cells, they can be expanded in the presence of estrogen while taking away estrogen, i.e., turning off Hoxb8, induces their differentiation. Depending on the growth factor added during initial establishment of the progenitor lines, cells can be generated that are committed to macrophage or neutrophil lineages [15] or that maintain both myeloid and lymphoid differentiation potential (termed multipotent progenitor (MPP) cells or lymphoid-primed MPP (LMPP) cells [13]; these cells are expanded in FLT3-ligand (FLT3L) and will here be referred to as FL-P cells).

We established FL-P cell lines, wt and with deficiency in pro-apoptotic Bcl-2-family proteins, by transducing bone marrow from the respective gene-deficient mice with the Hoxb8-ER construct and expanding the cells in medium containing FLT3L. We established FL-P lines deficient in Noxa, in Bim or in Bim plus one of the BH3-only proteins Puma, Noxa or Bmf. The anti-apoptotic proteins Bcl-X_L_, Bcl-2 and Mcl-1 were easily detectable in the cell lines, and there was little difference between the cells of the various genotypes with the exception of higher Mcl-1-levels in Noxa-deficient cells (Supplementary Fig. S1A). These higher levels were seen in both Noxa-single and Bim/Noxa-double-deficient cells and were not surprising because Noxa is a known antagonist of Mcl-1 and induces its proteasomal degradation [16]. Bim was easily detectable by western blotting, and the Bim levels in FL-P cells were slightly higher than in MEFs (Supplementary Fig. S1B), while Puma was clearly more highly expressed in FLP cells than in MEFs (Supplementary Fig. S1C). Mouse Noxa is not easily detectable by western blotting but we did obtain a signal in FLP cells, where expression was somewhat lower than in committed Hoxb8 neutrophil progenitor cells (Supplementary Fig. S1D). We were unable to detect Bmf by western blotting in LMPP. FL-P cells underwent apoptosis upon treatment with the topoisomerase II-inhibitor etoposide. Bim-deficiency provided some protection, additional loss of Bmf had no effect but additional loss of Puma did. Noxa-deficient cells showed substantial protection, and Bim/Noxa double-deficient cells were strongly protected (Supplementary Fig. S2A, B). This pattern reflects the expected roles of BH3-only proteins in DNA-damage induced apoptosis. The loss of either Bax or Bak provided no protection but in the absence of both Bax and Bak, cells were completely protected against apoptosis (evidenced by the appearance of active caspase-3) and cell death (Supplementary Fig. S2C–F).

**Fig. 1:**
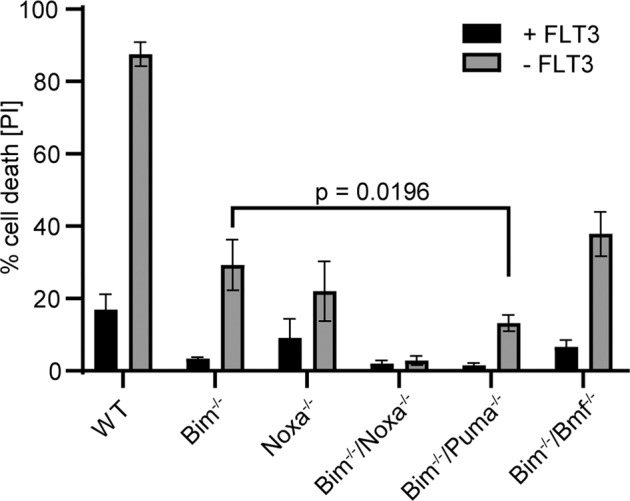
Role of BH3-only proteins in factor-withdrawal-induced apoptosis in FL-P cells. FL-P cells of the indicated genotypes were incubated in the presence or absence of growth factor (FLT3L) for 14 h, and cell viability was assessed by propidium iodide staining and flow cytometry. Shown are the mean values/SD of dead cells in three independent experiments; significance was tested by unpaired *t*-test.

FL-P cells are cultured in FLT3L and die rapidly upon its withdrawal. Wt cells showed about 50 % of cell death at 8 h and 90 % cell death by 14 h post-withdrawal (Fig. 1, Fig. S2G, H). Bim-deficient cells were substantially protected. Additional lack of Bmf afforded no additional protection. Loss of Puma on a Bim-deficient background did provide additional protection. Isolated loss of Noxa reduced apoptosis strongly, with cells deficient in Bim and Noxa showing hardly any apoptosis upon factor-withdrawal (Fig. 1). Upon growth factor withdrawal, Mcl-1-levels decreased. In the absence of Noxa, Mcl-1-protein started at higher levels and did not disappear as quickly. There was no clear increase in Bim- and a small, inconsistent increase in Puma-levels in these experiments (Supplementary Fig. S3). As has been recognized for other immune cell populations, Bim is thus important in FL-P cells but Noxa makes a substantial contribution to apoptosis in the situations tested here, and this Noxa-effect likely operates through antagonism of Mcl-1.

### The role of BH3-only proteins in cell death induced by Bcl-2-family inhibitors

**Fig. 2:**
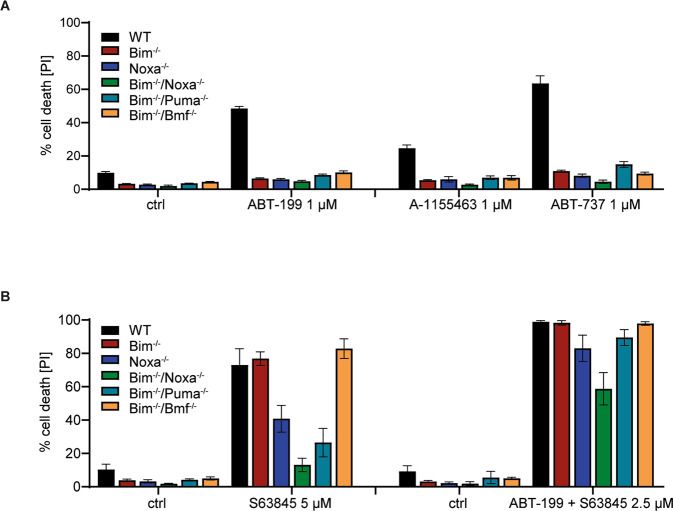
Importance of individual BH3-only proteins in apoptosis induced by inhibition of anti-apoptotic Bcl-2-family proeins. FL-P cells of the indicated genotypes were treated with the indicated Bcl-2-family inhibitors at different concentrations or combinations for 24 h as indicated; controls were cells incubated with equal concentrations of solvent (DMSO). In the combination with S63845, ABT-199 was used at 1 µM. Cells were analyzed by staining with propidium iodide and flow cytometry, and percentages of dead cells were calculated. Data are means/SD of three (**A**) or five (**B**) independent experiments. Data are individual points of the titrations shown in Supplementary Fig. S4.

We inhibited individual Bcl-2-proteins alone or in combination and tested the role of BH3-only proteins. A number of small molecules have been developed, which variably inhibit the main anti-apoptotic proteins. The Bcl-2-specific inhibitor ABT-199 [17] showed high activity and induced cell death at low concentrations (about 40 % cell death at 50 nM; Fig. 2, Supplementary Fig. S4), but higher concentrations had only very moderate additional effects. Only at concentrations of 2–10 µM, cell death increased further substantially (Supplementary Fig. S4A). The pro-apoptotic activity at concentrations up to 2 µM was almost entirely lost in the absence of either Bim or Noxa. At high concentrations, cells of all genotypes died although there was still reduced sensitivity in cells lacking the combination of either Bim and Noxa or Bim and Puma. Cell death completely depended on the presence of either Bax or Bak as cells double deficient in Bax and Bak were protected (Supplementary Fig. S4A). It seems likely that ABT-199 at high concentrations also neutralizes other anti-apoptotic Bcl-2-family proteins, as has been shown for Bcl-X_L_ [17]. Inhibition of Bcl-X_L_ (A-1155463 [18]) had only a small effect (Fig. 2A). The combination of Bcl-X_L_-inhibitor and ABT-199 (together inhibiting both Bcl-2 and Bcl-X_L_) was roughly additive (Supplementary Fig. S2B), and a very similar result was seen when both anti-apoptotic proteins were inhibited with ABT-737 (Fig. 2,Supplementary Fig. S4C). In all cases, the loss of Bim almost completely protected against apoptosis induced by these inhibitors (Fig. 2, Supplementary Fig. S4; the effect of Bim-loss at later time points is shown in Supplementary Fig. S4H–K). The detection of cells harboring active caspase-3 upon inhibitor treatment correlated with annexin V/PI-staining, and the combined loss of Bax and Bak abrogated the appearance of both stains (Supplementary Fig. S5). There was no appreciable change in the levels of the detectable anti-apoptotic Bcl-2-family proteins during treatment with ABT-737 (not shown). Blocking Bcl-2 and Bcl-X_L_ (and Bcl-w) at the same time therefore is a strong pro-apoptotic stimulus in FL-P, with Bcl-2 being more important. This form of apoptosis critically depends on both Noxa and Bim. This suggests that Bim is in these cells normally sequestered by mostly Bcl-2 and to a lesser degree by Bcl-X_L_. Noxa most likely acts by regulating the abundance of Mcl-1 [19]: in the absence of Noxa, active Bim is probably not sufficient to overcome the anti-apoptotic effect of Mcl-1.

**Fig. 3:**
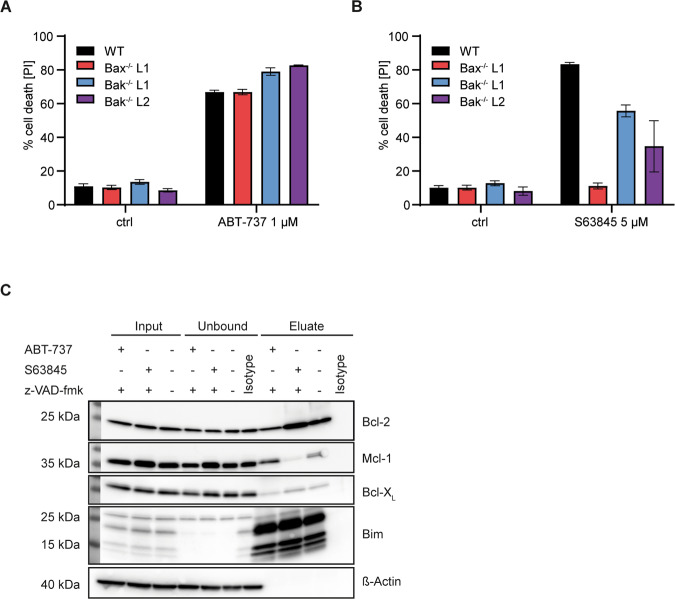
Role of Bax vs. Bak and interactions of Bim in FL-P cells. **A**, **B** FLP-cells of the indicated genotypes were treated with Bcl-2-family inhibitors at the concentrations indicated (controls were incubated with DMSO) for 24 h and analyzed by propidium staining and flow cytometry. Shown are mean values/SD of three independent experiments. Data are individual points of the titrations shown in Supplementary Fig. S4. **C** Immunoprecipitation of Bim and Bim-interacting Bcl-2-family proteins. FL-P cells (wt) were incubated with 1 µM ABT-737 or 3 µM S63845 plus 20 µM z-VAD-fmk or DMSO for 4 h. Input and unbound are equivalent protein amounts; eluate was the complete IP-product. Proteins were separated by SDS-Page and transferred to a PVDF membrane. Membranes were probed for Bcl-2 proteins as indicated. Blots are representative of three independent experiments.

Because at least most mammalian cells die when Bcl-2/Bcl-X_L_ and Mcl-1 are inhibited, this suggested that an essential function of Bim was the neutralization of Mcl-1, upon displacement from Bcl-2. Consistent with this interpretation, inhibition of Mcl-1 (using S63845 [20]) induced cell death that was Bim-independent. Loss of Noxa provided some protection, presumably through the higher Mcl-1-levels, and the combined loss of Bim and Noxa or Bim and Puma protected the cells substantially (Fig. 2B, Supplementary Fig. S4D). Combination treatments with ABT-199/737 and S63945 were also consistent with the interpretation that Bim was required for Mcl-1-neutralization: increasing concentrations of the Mcl-1-inhibitor increasingly removed the requirement for Bim (Fig. 2B, Supplementary Fig. S4E). We further tested for the individual requirement of Bax and Bak. Upon ABT-737-treatment, loss of either alone provided no protection (Fig. 3A). Because this form of apoptosis depends on Bim, this suggests that Bim can in these cells activate both proteins, either through antagonizing Mcl-1 or through direct activation. Intriguingly, Mcl-1-antagonism-induced apoptosis received some contribution from Bak but was entirely dependent on Bax (Fig. 3B, Supplementary Figs. S4F, G, S4H–M for later time points). In this situation, Noxa contributes suggesting that Mcl-1 can block both Bax and Bak. Because this form of apoptosis also requires Puma (presumably together with Bim), the results further indicate that Puma can activate Bax but not Bak in this situation; this may be the result of a reduced availability or ability of anti-apoptotic proteins to inhibit Bax.

We then tested binding of Bim to anti-apoptotic Bcl-2-proteins by immunoprecipitation. Bim was precipitated efficiently; although Mcl-1, Bcl-2 and Bcl-X_L_ were all found in the IP-product to some extent, a very substantial part of these proteins remained in the supernatant (Fig. 3C). Inclusion of ABT-737 or S63845 shifted Bim away from the respective targets and to the untargeted binding partners although this shift was incomplete (Fig. 3C).

### Dynamics of Bcl-2-family expression during early B cell differentiation

**Fig. 4:**
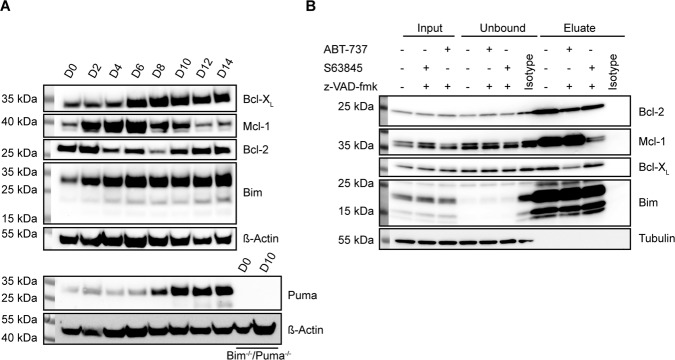
Changes in expression of Bcl-2-family proteins and Bim-interactions during differentiation of early B cells. **A** Western blot analysis of endogenous Bcl-2 protein levels in wt Hoxb8-FLT3 cells differentiating in vitro. FL-D cells were cultured in the presence of IL-7, FLT3L and SCF for 14 days. Samples were taken every two days, lysed and analyzed by SDS-PAGE and western blotting. Membranes were probed for Bcl-2 family proteins as indicated. Shown are two different membranes from the same cell lysates. **B** Analysis of Bim-interacting Bcl-2-family proteins. FL-D cells (differentiated for 14 d as above) were incubated with either 1 µM ABT-737 or 3 µM S63845 in the presence of 20 µM z-VAD-fmk or DMSO. Bim was immunoprecipitated (isotype, isotype control antibody). Samples were processed for western blotting. Input and unbound are equivalent protein amounts; eluate was the complete IP-product. Membranes were probed for Bcl-2 proteins as indicated. All blots are representative of three separate experiments.

We used the Hoxb8-model to differentiate the LMPP (FL-P) cells to early B cells (here termed FL-D cells). Because it was difficult to separate the cell populations for our purposes, we replaced the OP9-feeder cells of the original protocol [13] with the soluble factors FLT3L, IL-7 and SCF. When transplanted into mice, FL-P-cells differentiate into fully mature B cells that have been re-isolated from the spleen. By about day 11 after Hoxb8-inactivation, most cells expressed markers of pro-B cells (CD19^+^B220^+^CD93^+^CD24^+^) as well as the surrogate light chains (CD179a/b); some cells also expressed BP-1 (early pre B cells) but not intracellular heavy chain. Developing B cells undergo heavy chain rearrangement during the transition to pro-B cells and VDJ-rearrangement before becoming pre-B cells. The cells we use here were mostly at the pro-B cell stage. There was no major phenotypic change after day 11 (Supplementary Fig. S6) but the cells kept expanding. The expression of Bim was strongly up-regulated during the first 4 days and remained at comparably high levels from then on (Fig. 4A). Puma was strongly up-regulated around day 10 (late pro B cell stage). The expression of Bcl-X_L_ was relatively low initially and increased substantially between days 4 and 6. Great dynamic was however seen in the regulation of Mcl-1- and Bcl-2-expression. Both proteins showed similar expression levels at progenitor stage and after about 12 days of differentiation. In between, however, here was a drastic up- and down regulation of Mcl-1 protein between days 2 and 6. Bcl-2, on the other hand, was transiently down-regulated between days 4 and 8 (Fig. 4A). The regulation of Mcl-1 was also seen in Noxa-deficient cells (Supplementary Fig. S7A). We were unable to obtain a clear staining for Mcl-1, but intracellular staining and flow cytometry of murine bone marrow confirmed differences in Bcl-2-expression between the various stages of B cell-development (Supplementary Fig. S7B).

### Role of Bcl-2-family members in early B cells

**Fig. 5:**
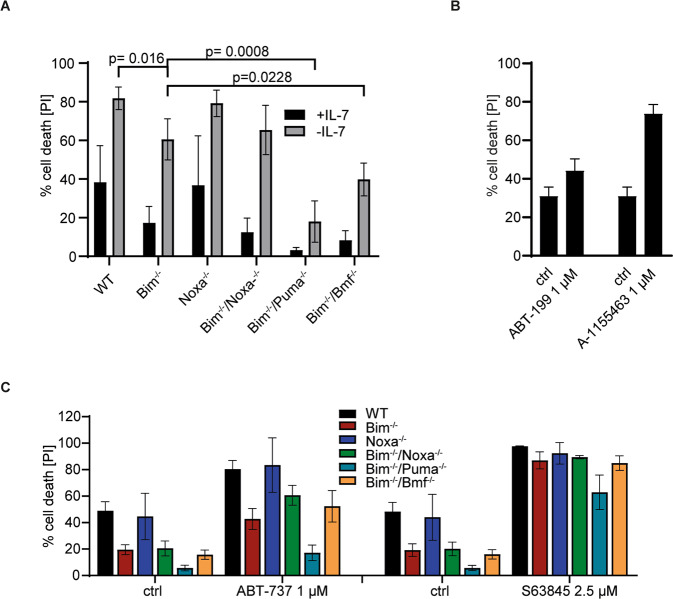
Role of BH3-only proteins in apoptosis induced by inhibition of anti-apoptotic Bcl-2-proteins in FL-D cells. **A** FL-D cells (10^5^ d14 in vitro differentiated cells) of the indicated genotypes were incubated in presence or absence of IL-7. Cell viability was determined after 24 h by propidium iodide staining and flow cytometry. Significance levels were calculated using unpaired *t*-test. **B** FL-D cells (10^5^ d14 in vitro differentiated cells) were treated with 1 µM ABT-199 or 1 µM A-1155463. Cell viability was determined after 24 h by propidium iodide staining and flow cytometry. Shown are the means/SD of three independent experiments. Data are taken from the complete titration shown in Supplementary Fig. S7. **C** FL-D cells (10^5^ d14 in vitro differentiated cells) of the indicated genotypes were treated with 1 µM ABT-737 or 2.5 µM S63845. Cell viability was determined after 24 h by propidium iodide staining and flow cytometry. Data are means/SD of three independent experiments. Data are individual points of the titrations shown in Supplementary Fig. S7. Supplementary Information is available at Cell Death and Differentiation’s website.

We differentiated the cells to test whether differentiation status (differentiation from the LMPP to the pro-B cell stage) affected the role of Bcl-2-proteins in apoptosis sensitivity. It was noticeable that in differentiated cells more anti-apoptotic protein was co-immunoprecipitated with Bim (Fig. 4B; compare IP-product with input in Fig. 4B and Fig. 3C), suggesting that Bim was increasingly complexed by anti-apoptotic Bcl-2-family proteins. We then tested apoptosis sensitivity in cells differentiated as above for 14 days (FL-D cells). Cells underwent rapid cell death upon IL-7-withdrawal. Loss of Bim provided some protection, as found previously for T cell progenitors [21]. Intriguingly, Noxa had lost all importance with regard to factor withdrawal during differentiation even on a Bim-deficient background, but Puma had acquired a prominent role: in the absence of Bim, Puma-deficiency provided strong protection, similar to the role previously identified in activated T cells [22]. The loss of Bmf on a Bim-deficient background also provided some protection (Fig. 5A).

Although Bcl-2-levels were only slightly reduced in FL-D compared to FL-P cells, Bcl-2-inhibition had little pro-apoptotic effect while inhibition of Bcl-X_L_ was a strong pro-apoptotic signal (Fig. 5B), reflected by a strong up-regulation of the protein (Fig. 4). Loss of Bim had some protective effect against ABT-737-induced cell death while Bim/Puma-deficiency gave strong protection (Fig. 5C). Cell death induced by Mcl-1-inhibition again did not depend on Bim, but the combined loss of Bim and Puma afforded some protection (Fig. 5C; Supplementary Fig. S7). Substantial changes in Bcl-2-protein function during differentiation from LMPP cells towards B cells thus include the loss of the importance of Noxa, the gain in importance of Puma, a somewhat smaller importance of Bim itself in ABT-737-induced apoptosis, and changes in the relevance of Bcl-2 and Bcl-X_L_.

## Discussion

Information about the relevance to apoptosis-induction in immune cells had been available for Bim and, in some situations, for other BH3-only proteins. Our results identify the importance of Bim specifically for the neutralization of Mcl-1 and assign functions to Noxa and Puma. They further describe unexpectedly strong shifts in the expression of especially anti-apoptotic Bcl-2-family proteins during early B cell differentiation as well as partly non-redundant roles of Bax and Bak. Stage-specific expression of Bcl-2-family proteins in culture suggests an intrinsic regulation and a specific role of the individual members of the family. By combining the loss of pro-apoptotic with the chemical neutralization of anti-apoptotic Bcl-2-family proteins, we obtained information about the roles of individual proteins in the network.

Bim is required for full apoptosis in most immune cells. Bim can principally neutralize all anti-apoptotic Bcl-2-proteins and activate Bax and Bak directly. What it actually does upon apoptosis initiation is however not clear. In FL-P cells, Bim was essential to apoptosis induced by inhibition of Bcl-2 and Bcl-X_L_. In this situation Bax and Bak were redundant, suggesting that Bim can activate either protein. Bim may here either directly activate Bax/Bak or inactivate Mcl-1. Although it is not possible completely to exclude either possibility, the finding that the addition of a Mcl-1-inhibitor induces cell death that is independent of Bim suggests that the function of Bim is the neutralization of Mcl-1. Isolated Noxa-deficiency had a surprisingly strong effect in FL-P cells. Noxa-deficient cells had more Mcl-1-protein, and the higher doses of Mcl-1-inhibitor could compensate for the loss of Noxa. This suggests that the role of Noxa indeed is the regulation of Mcl-1-levels. Mouse Noxa differs from human Noxa especially in that it has a second BH3-domain, although experiments with truncation mutants suggest that only the C-terminal BH3-domain is used to inactivate Mcl-1 [23]. There may be additional functional differences to human cells [24]. In the absence of Bim, Mcl-1-inhibition required Puma for efficient apoptosis-induction. Because of the critical role of Bax for apoptosis-induction by Mcl-1-inhibition, Puma – perhaps together with Bim – in this situation very likely can activate Bax. In activated T cells, Puma has been reported to have a pro-apoptotic role that is only detectable in the absence of Bim; in developing B cells, Bim- but not Puma-deficiency could to a degree rescue development. No information appears to be available in other cell types. More work will still be required to understand all the mechanics, but the results identify critical and redundant pairings in the interaction of Bcl-2-family proteins. The relative importance of anti-apoptotic proteins in keeping FL-P cells alive runs as Mcl-1-Bcl-2-Bcl-X_L_.

An intriguing result of the IP-experiments was that, despite the relevance of Bim for apoptosis-induction, relatively little of the anti-apoptotic Bcl-2-proteins were co-precipitated with Bim; the inclusion of ABT-737 caused no visible change in the abundance of Bcl-2 or Bcl-X_L_ in the fraction not bound to Bim (although some change was seen for Mcl-1). We have to allow for experimental imperfections in the sense that complexes may to some degree be sensitive to cell lysis and detergent. It seems a possibility however that most Bim is actually not bound to anti-apoptotic Bcl-2-proteins, and may indeed not be active. We have recently described that Bim can be found in large complexes coordinated by dynein light chain [25], and these complexes may represent inactive ‘stores’ of Bim. Isolation and characterization of complexes is an important issue, which we have not fully addressed in this study. It is a limitation that we have so far only immuno-precipitated Bim. This showed the surprisingly low amount of binding to anti-apoptotic proteins. Reciprocal IPs, for instance of Mcl-1, will be helpful to understand complex formation within the Bcl-2-family better.

It is notoriously difficult to understand the manifold interactions of pro- and anti-apoptotic Bcl-2-family members and how they change during the induction of apoptosis, either through upstream signals such as factor withdrawal or through the specific inhibition of Bcl-2-like proteins. Approaches such as quantitative proteomic analysis of precipitates and cross-linking mass-spectrometry [26] will in the future be helpful to clarify how these interactions change and may even be able to elucidate whether a BH3-only protein acts through direct activation or through displacement [27]. It may for instance be possible to discover the shift of Mcl-1 (or other anti-apoptotic proteins) away from Bim to the binding of Bak.

Comparing early B cells to FL-P cells, Noxa had lost all importance during apoptosis induced by Bcl-2-family inhibition or factor withdrawal. Puma, on the other hand, had considerably gained in relevance, paralleled by the strong up-regulation of the protein. Mcl-1 still had the most important anti-apoptotic role but Bcl-X_L_ was now much more important than Bcl-2. Bim itself was expressed more highly in FL-D cells but its importance in apoptosis induction appeared somewhat smaller than its role in FL-P cells.

Bmf, finally, is a BH3-only protein that has, based on experiments with gene-deficient cells, only a limited role in the cells investigated so far, with the most prominent role reported in B cells [9]. Our results confirm this role: an effect of Bmf-loss (in addition to Bim) on factor-induced apoptosis was seen in early B cells but not in LMPP. Bmf however played no role in regulating apoptosis induced by Bcl-2-family-inhibitors. Because of the independence of anti-apoptotic proteins, this is most easily explained by an upstream regulatory effect of factor-withdrawal on Bmf-activity.

One of the striking changes during early B cell differentiation was the transient upregulation of Mcl-1. Mcl-1-expression during B cell development has been measured before on the mRNA-level [28], and no similar change had been observed. Because Mcl-1 is strongly regulated by post-translational signals [29], this is no contradiction but suggests that the effect is due to signals that regulate protein stability. During differentiation in the Hoxb8-model the external stimuli do not change during culture. The data therefore suggest that Mcl-1-regulation occurs as part of the intrinsic differentiation program. A likely signaling pathway is the PI3 kinase/AKT signaling axis, which is a well-established regulator of Mcl-1-stability [30], and which is active during early B cell development [31]. The signals regulating expression of the other Bcl-2-family proteins during B cell development are less clear, but our findings suggest regulation of these proteins through signals that are generated cell autonomously as part of the B cell maturation program. A further intriguing aspect is the difference in susceptibility of FL-P and early B cells when growth factors are removed: in FL-P cells, Noxa plays an important role, in the early B cells Puma was much more important. The different factors to which the cells are ‘addicted’ may play a role. As in many cases of upstream signals, it is only partially clear how a signal from cytokine receptors, or the signal of their absence reaches mitochondria. Most consistent has been the description of the regulation of Mcl-1-levels through this axis, and indeed this has been found for both FLT-3 ligand [32] (which we used for the FL-P cells) and IL-7 (or other signals through the common gamma chain) [33], the survival stimulus in our B cell cultures. Mcl-1-regulation itself is very complex and involves transcriptional and post-translational modifications [34]. Noxa is one major regulatory molecule of Mcl-1-levels [16], so the regulation of cell death upon FLT3-ligand-withdrawal in FL-P cells, where Noxa plays a major role, likely occurs through this pathway. How apoptosis is regulated upon withdrawal of IL-7 is unclear. Mcl-1 may be degraded by numerous other mechanisms, and this may for instance allow the activation of Bak [35]. Differences and potential common regulation in these situations will require a more detailed study. The significant role of Puma in apoptosis-induction in early B cells but not in FL-P cells correlated with its substantial upregulation. This regulation is intriguing: Puma is best known as a p53-target [36, 37] but can also be regulated by other transcriptional mechanisms. NF-κB in particular can drive Puma transcription [38] and is a likely contributor during B cell development.

Through the large number of proteins, the differential expression and over-lapping functions, the regulation of Bcl-2-protein activity is extremely challenging to map. Our results add information about the molecular role of these proteins in the orchestration of apoptosis in a cellular model that probably reflects differentiation stages of murine lymphoid cells. To some extent, they suggest new hypotheses that will need to be tested. By the broad testing of pro-apoptotic proteins through gene-deficiency, combined with the specific inhibition of anti-apoptotic Bcl-2-proteins, they identify molecular roles of individual proteins as well as combinations of family members. The knowledge of the roles of individual Bcl-2-family proteins in lymphoid progenitor cells may inform studies of Bcl-2-family inhibitors on apoptosis induction in malignant lymphoid cells and eventually the use of these inhibitors in the clinic [39].

## Material and methods

### Generation of FLT3-driven LMPP lines

LMPP cell lines (FL-P) were established from mouse bone marrow of the respective gene-deficient mice as reported [13] (the single-deficient mice have been described [6, 9, 40, 41]. The crosses of mice deficient in Bim and other BH3-only proteins were conducted by Dr Andreas Villunger, Innsbruck, who kindly provided the bone marrow). Briefly, bone marrow cells were infected with a retrovirus expressing oestrogen-regulated Hoxb8 (generously provided by Hans Häcker, Salt Lake City). Cells were expanded in the presence of oestrogen and FLT3L. For Bim-deficient cells, several cell lines were initially established from several mice and tested for differences in survival and differentiation; no substantial differences were seen. Cells were cultured in VLE RPMI medium supplemented with 10% FCS (Gibco), 1% Pen-Strep (), 1% GlutaMax (Thermo Fisher), 50 µM ß-mercaptoethanol (Gibco), 10 µM β-estradiol (Sigma Aldrich) and 5 % FLT3L-containing supernatant from a transgenic B16 mouse melanoma cell line

### In vitro differentiation of early B cells

Hoxb8 FLP cells were washed twice in PBS with 1% FCS to remove β-estradiol. Cells (4 × 10^5^ cells in 2 ml in 6-well plates) were resuspended in B-cell medium: DMEM, high glucose, (Gibco) + 10% FCS (Gibco) + 50 µM ß-mercaptoethanol (Gibco) + 1 % Pen-Strep (Gibco) + 1% sodium pyruvate (Gibco) + 15 ng/ml recombinant murine IL-7 (Peprotech) + 30 ng/ml recombinant murine FLT3L + 7.5% CHO-SCF-containing supernatant and grown for various times. Cells were resuspended in fresh medium on day 2, split into two wells on day 3, then split every 2–3 days with addition of fresh medium. For cell death assays, cells were harvested on day 14 of differentiation. Live cells were separated using the MACS Dead Cell Removal kit (Miltenyi Laboratories).

### Cell death assays

FL-P or FL-D cells were plated in 24-well plates (10^5^ cells in 500 µl Hoxb8 progenitor or B cell medium) and treated with etoposide (Sigma Aldrich) or the inhibitors ABT-737, ABT-199, A-1155463 (Selleckchem) or S53845 (ApexBio) as indicated. For analysis of cell death induced by factor withdrawal, cells were washed and plated without FLT3L (FL-P-cells) or IL-7 (FL-D-cells). At various time points, cells were collected in PBS/4% FCS. Propidium iodide (Sigma Aldrich) was added immediately prior to analysis by flow cytometry (FACS-Calibur, Becton Dickinson). In some experiments, annexin V-propidium iodide staining was used for quantification of apoptosis. Cell were washed with annexin V-binding buffer (eBioscience) and stained with annexin V-FITC (1:20, BD Pharmingen) and PI (5 µg/ml) for 20 min at 4 °C followed by flow cytometry analysis (FACS Calibur). For staining of active caspase-3, cells were fixed in 2% paraformaldehyde and permeabilized with 0.5% saponin (Sigma-Aldrich). Cells were incubated with anti-active caspase-3 (BD Pharmingen) in PBS/0.5%BSA/0.5% saponin for 30 min, stained with anti-rabbit-Alexa-Fluor488 (Dianova GmbH, Hamburg, Germany) for 30 min and analyzed by flow cytometry (FACS Calibur). Routinely, three biological replicates were performed. Further replicates were done depending on the statistical distribution of the values.

### Western blotting

FL-P or FL-D cells (5 × 10^6^) were washed and lysed in 100 µl lysis buffer BOLT lysis buffer, sonicated and loaded on BOLT 4–12% Bis-Tris precast gels (ThermoFisher). Proteins were transferred to nitrocellulose membranes by wet transfer, and specific proteins were detected with antibodies against Bim (Cell Signaling, clone C3435), Noxa (Abcam, polyclonal), Puma (Cell Signaling, polyclonal), Mcl-1 (Rockland, polyclonal and BioLegend, W16014A [42]), Bcl-2 (BioLegend, 3F11), Bcl-X_L_ (Cell Signaling, 54H6), ß-Actin (Sigma Aldrich, AC-15), GAPDH (Millipore, 6C5), α-Tubulin (Sigma Aldrich, DM 1A). Secondary antibodies used were goat-anti-rabbit (Millipore), goat-anti-mouse Millipore or goat-anti-rat (Cell Signaling) coupled to peroxidase. Signals were detected with ECL Pico/Prime/Femto using a chemiluminescence detection system (Intas Detection Systems).

### Immunoprecipitation

Cell pellets (4.5 × 10^7^ cells per sample) were lysed in 750 µl lysis buffer: 20 mM Tris-HCl (Tris, Sigma Aldrich; HCl, Carl Roth), 150 mM NaCl (Carl Roth), 10% Glycerol (Carl Roth), 1% Triton X-100 (Sigma Aldrich), 1x protease inhibitor cocktail (Roche) on ice and centrifuged for 10 min at 10.000 × *g*. Supernatants were transferred to a new tube. Protein concentrations were measured by Braford assay (BioRad), and 1500 µg of protein were used for IP. Bim-antibody (Cell Signaling, C3435), or isotype control antibody (mouse mAb IgG1κ, 2.5 mg/ml) Cell Signaling, G3A1) was added to protein and Agarose G beads (Millipore Sigma, 30 µl slurry per IP). Samples were incubated for 4 h at 4 °C with overhead rotation. After incubation, reactions were centrifuged, unbound fraction was collected. IP-products were washed 1x with 15 ml washing buffer and subsequently washed three times with 350 µl washing buffer. Proteins were eluted by boiling in 3x Laemmli buffer (95 °C, 5 min). For input and unbound samples, 50 µg samples (for unbound fraction, 1/30) were used per lane. Samples were run on SDS-PAGE as above and blotted onto PVDF-membranes. Detection was carried out as described above.

### Flow cytometry

For differentiation of differentiating cells, cells were harvested, washed and stained with the following antibodies: CD44-APC (BD, IM7), CD25-FITC (eBioscience, PC61.5), CD93-APC (eBioscience, AA4.1), B220-FITC (BD, RA3–6B2), CD19-APC (eBioscience, 1D3), IgM-PE (eBioscience, eB121–15F9), B220-APC (eBioscience, RA3–6B2), Thy1.2-FITC (eBioscience, 30-H12), CD135-APC (BioLegend, A2F10), CD127-PE (eBioscience, A7R34), BP-1-PE (eBioscience, 6C3), CD24-APC-Cy7 (eBioscience, M1/69), CD43-APC (BioLegend, S11). For intracellular staining, cells were permeabilized using 0.05 % saponin and stained with antibodies against CD79a-APC (BioLegend, F11–172), CD79b-FITC (BioLegend, HM79–12), CD179a-BV421 (BioLegend, R3), CD179b-BB700 (BioLegend, LM34). For the Bcl-2-specific stain, the Foxp3 staining kit (ThermoFisher) was used. As antibody, Bcl-2-AF647 (BioLegend, 10C4) was used. Analysis was carried out using a FACS Canto II cytometer (Beckton Dickinson).

## Supplementary information

Supplemental figures S1-S8
