# Supplementary material for: Importance of Bcl-2-family proteins in murine hematopoietic progenitor and early B cells
Source: Cell Death Dis. 2021 Aug 11;12(8):784. doi: 10.1038/s41419-021-04079-8 (PMC8358012; doi:10.1038/s41419-021-04079-8)
Supplement: Supplementary file 1 — Supplemental figures S1-S8 [file 41419_2021_4079_MOESM1_ESM.pdf]

**Figure S1**

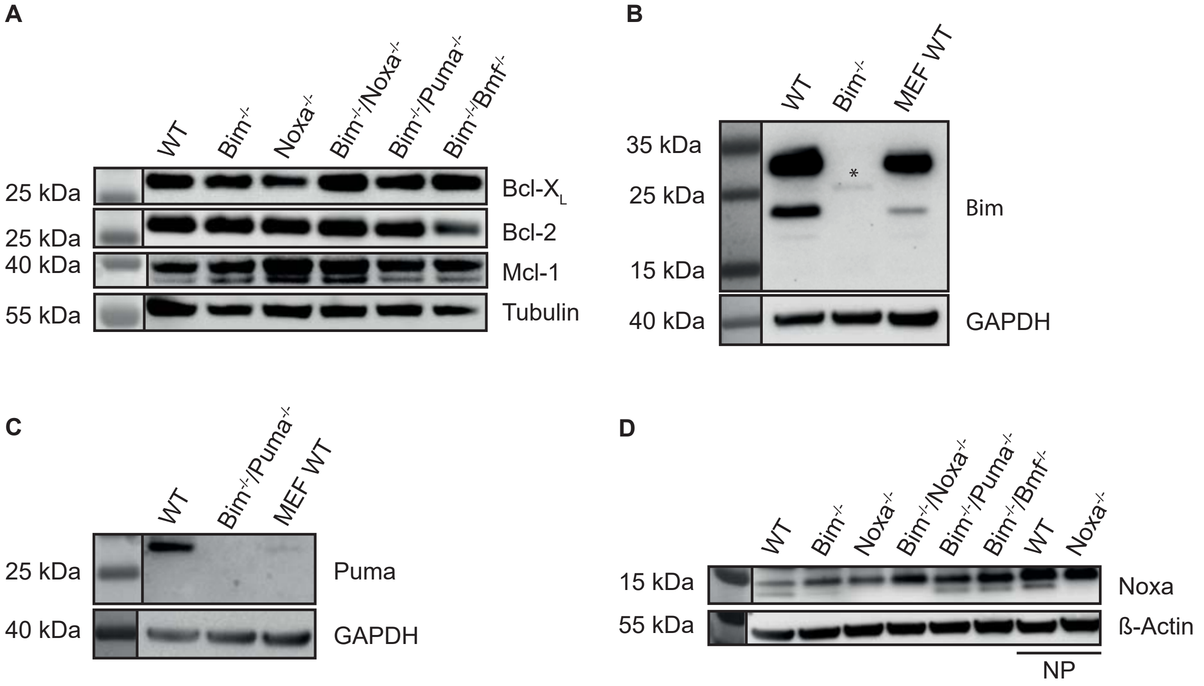


Western blot analysis of FL-P cells to determine (A) endogenous anti-apoptotic Bcl-2 protein levels, levels of (B) Bim, (C) Puma and (D) Noxa. Asterisk in (B) indicates a non-specific band. MEF wt cells were tested for comparison. (D), NP, Hoxb8 neutrophil progenitor cells (wt and Noxa-deficient [26]) were included for comparison. 5x $10^6$ cells of the indicated genotypes were lysed in 100 µl lysis buffer. Lysate (35 - 45 µl) was run on SDS-Page and transferred to a nitrocellulose membrane. All blots are representative for three independent experiments.

**Figure S2**

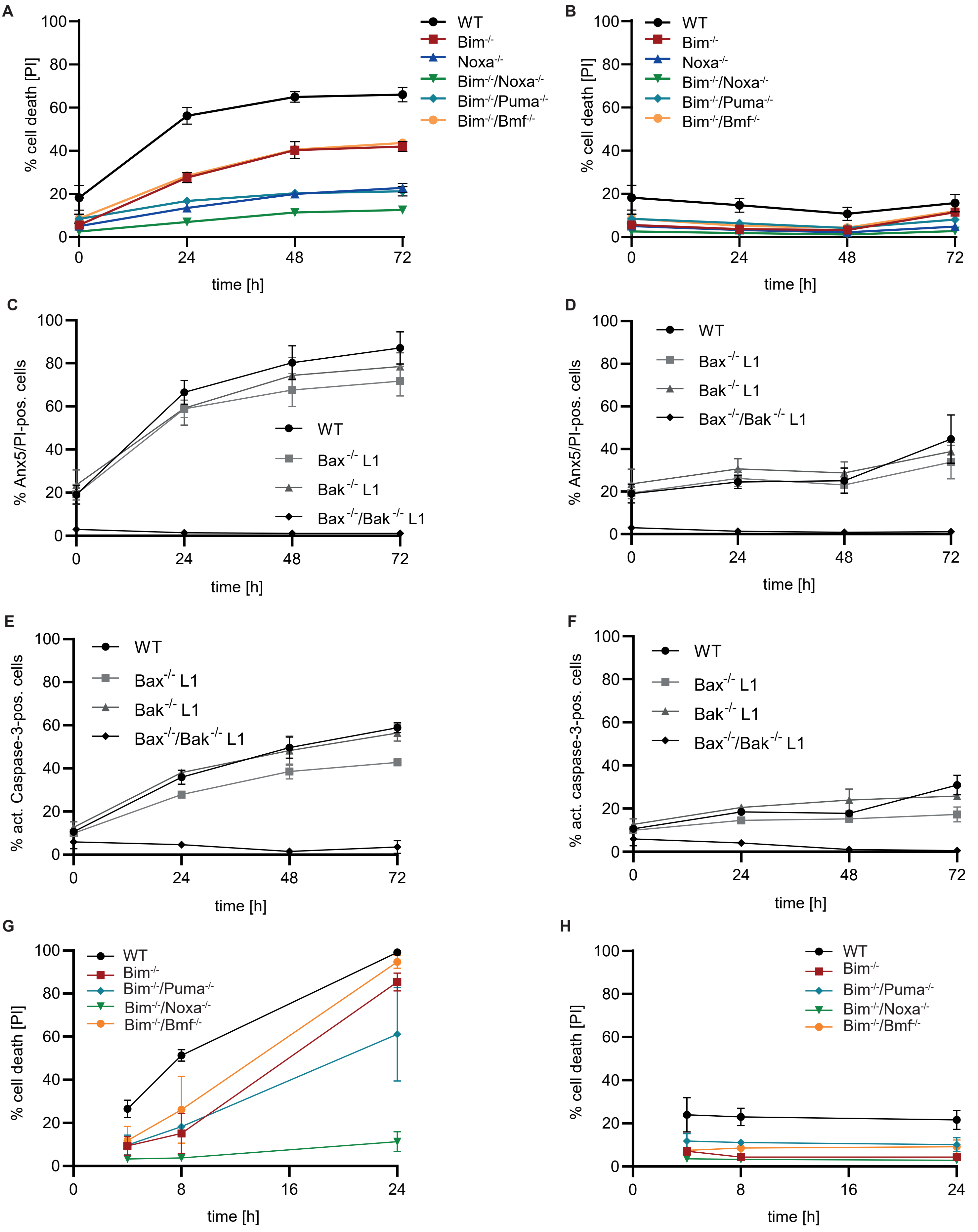


Figure S2:
A-F: FL-P cells of the indicated genotypes were treated with etoposide (150 nM) (A,C,E) or DMSO control (ctrl) (B,D,F) and analysed at 24, 48 and 72h. Cell viability was determined by propidium iodide (A,B), AnnexinV/PI (C,D) or active Caspase-3 (E,F) staining and flow cytometry. Data are means/SD of three independent experiments each.
G,H: FL-P cells of the indicated genotypes ($1x10^5$ cells in 24-well plates) were incubated in the presence (C) or absence (D) of growth factor (FLT3L) for 4, 8 and 24h, and cell viability was assessed by propidium iodide staining and flow cytometry.
Data are means/SD of three independent experiments.

**Figure S3**

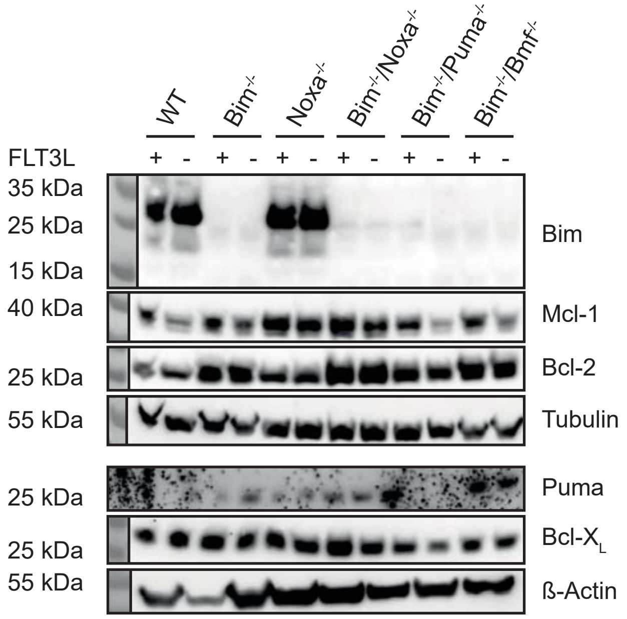


Figure S3: FL-P cells were incubated in the presence or absence of FLT3L for 4h. Cells of the indicated genotypes (5x $10^6$ in 100 µl lysis buffer) were harvested and subjected to Western blotting. Membranes were probed for Bcl-2 family proteins as indicated. Shown are two different membranes from the same lysates. The experiment was done twice with similar results.

**Figure S4**

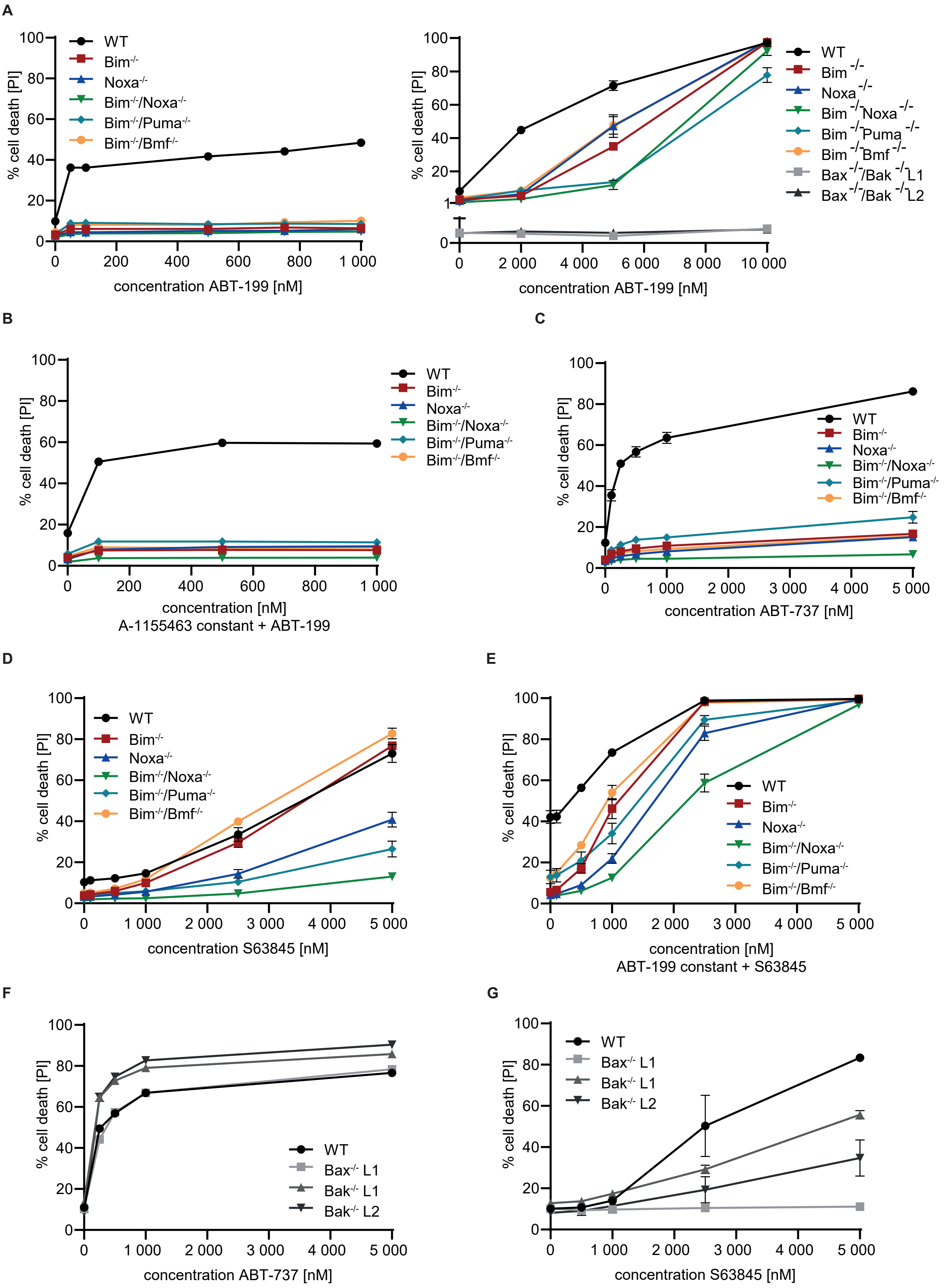
A
% cell death [PI]
concentration ABT-199 [nM]
WT
Bim-/-
Noxa-/-
Bim-/-/Noxa-/-
Bim-/-/Puma-/-
Bim-/-/Bmf-/-
Bax-/-/Bak-/- L1
Bax-/-/Bak-/- L2
B
% cell death [PI]
concentration [nM]
A-1155463 constant + ABT-199
C
% cell death [PI]
concentration ABT-737 [nM]
D
% cell death [PI]
concentration S63845 [nM]
E
% cell death [PI]
concentration [nM]
ABT-199 constant + S63845
F
% cell death [PI]
concentration ABT-737 [nM]
Bax-/- L1
Bak-/- L1
Bak-/- L2
G
% cell death [PI]
concentration S63845 [nM]

**Figure S4**

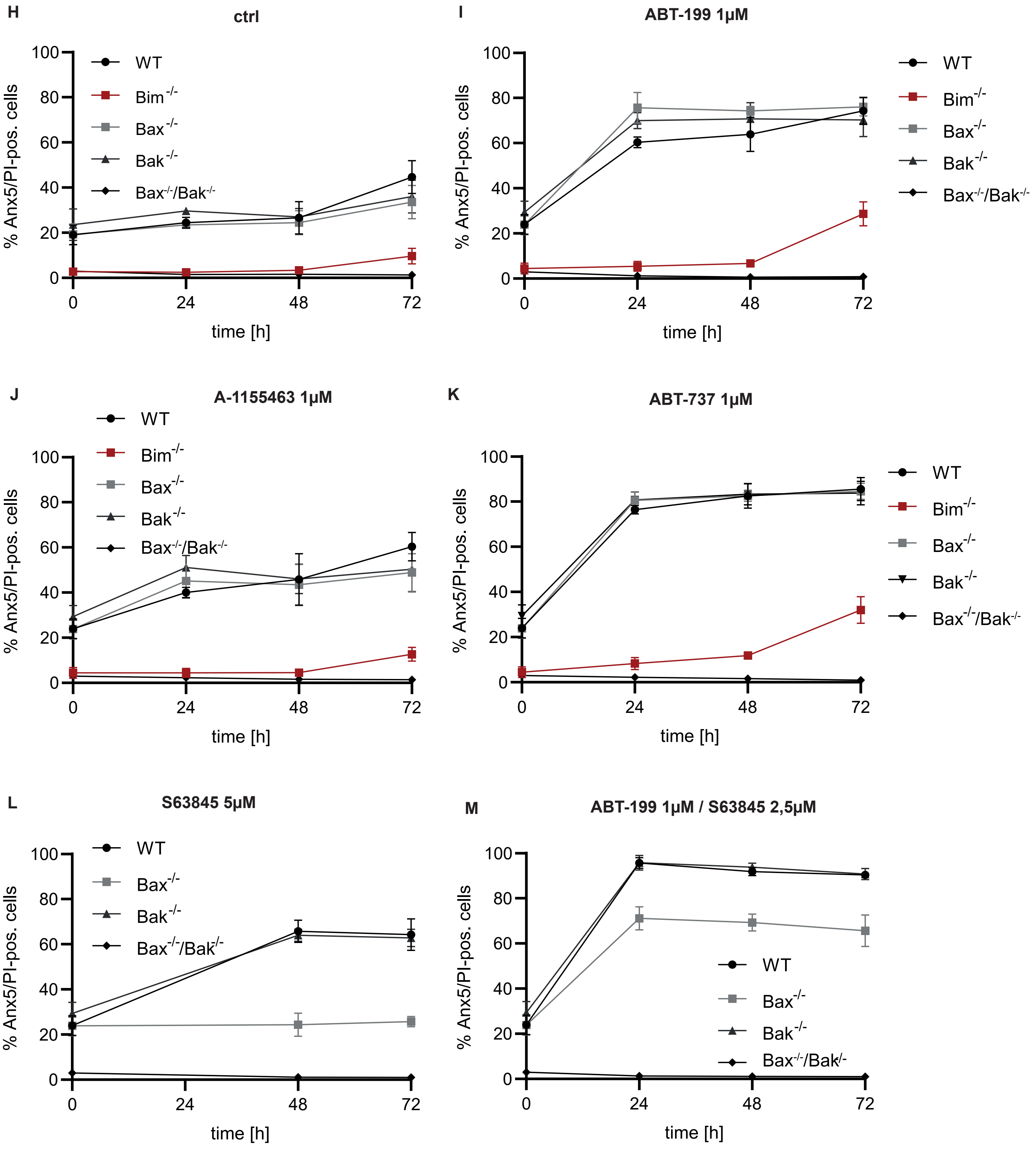


Figure S4: A-G, FL-P cells of the indicated genotypes ($10^5$ cells per well in 24-well plates) were incubated with increasing concentrations of ABT-199 (A), 1 µM of A-1155463 plus increasing concentrations of ABT-199 (B), increasing concentrations of ABT-737 (C, F) or S63845 (D, G), or 1 µM ABT-199 plus increasing concentrations of S63845 (E). Cell viability was determined after 24h by propidium iodide staining and flow cytometry. Data are means/SD of three (A, C, F, G), three to four (B) and five (D, E) independent experiments. A, F, G: L1 and L2 are two independently established lines.
H-M, FL-P cells of the indicated genotypes ($10^5$ cells per well in 24-well plates) were treated with Bcl-2-family inhibitors at defined concentrations or combinations as indicated for increasing time periods (0-72h); controls were incubated with equal concentrations of DMSO as solvent (ctrl). Cell viability was determined by annexinV/PI staining and flow cytometry. Data are means/SD of three independent experiments.

**Figure S5**

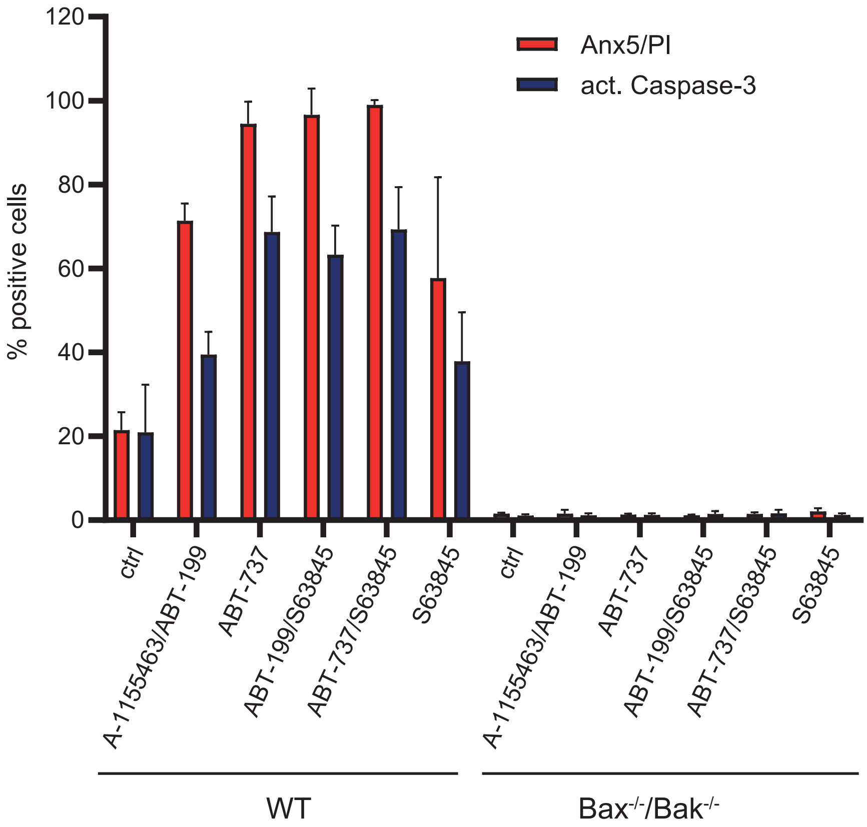


Figure S5: WT or $Bax^{-/-}/Bak^{-/-}$ (Line 1) FL-P cells ($10^5$ cells per well in 24-well plates) were incubated with the following concentrations of inhibitors either alone or in combination as indicated: ABT-199 1µM; A-1155463 1µM; ABT-737 10µM; S63845 5µM. Cell viability was determined after 24h by AnnexinV/PI staining (red bars) or staining against active Caspase-3 (blue bars) followed by flow cytometry. Data are means/SD of three independent experiments.

**Figure S6**

**A**

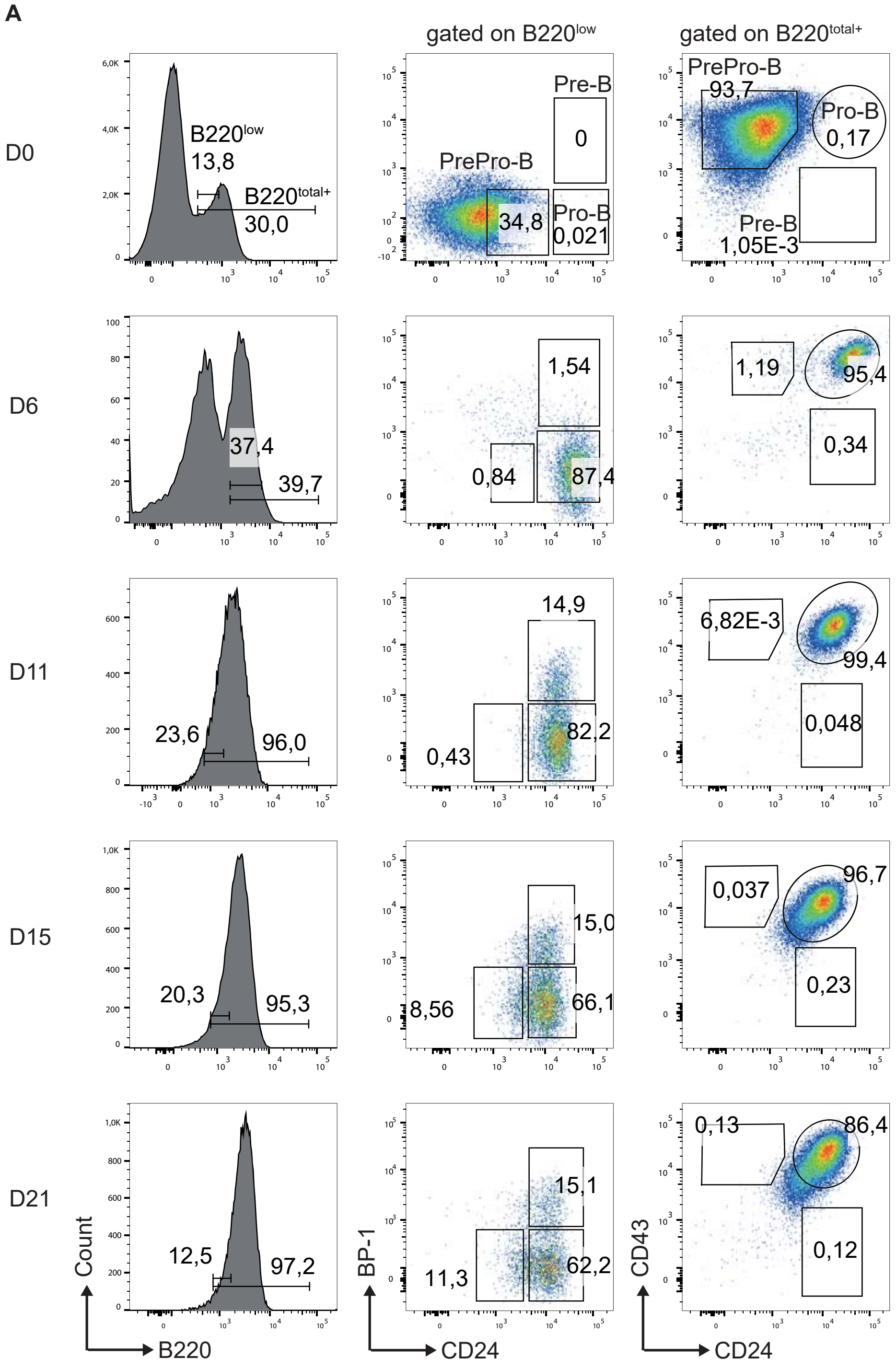
gated on B220low
gated on B220total+
D0
B220low
13,8
B220total+
30,0
Pre-B
0
PrePro-B
34,8
Pro-B
0,021
PrePro-B
93,7
Pro-B
0,17
Pre-B
1,05E-3
D6
37,4
39,7
1,54
0,84
87,4
1,19
95,4
0,34
D11
23,6
96,0
14,9
0,43
82,2
6,82E-3
99,4
0,048
D15
20,3
95,3
15,0
8,56
66,1
0,037
96,7
0,23
D21
12,5
97,2
15,1
11,3
62,2
0,13
86,4
0,12
Count
B220
BP-1
CD24
CD43
CD24

**B**

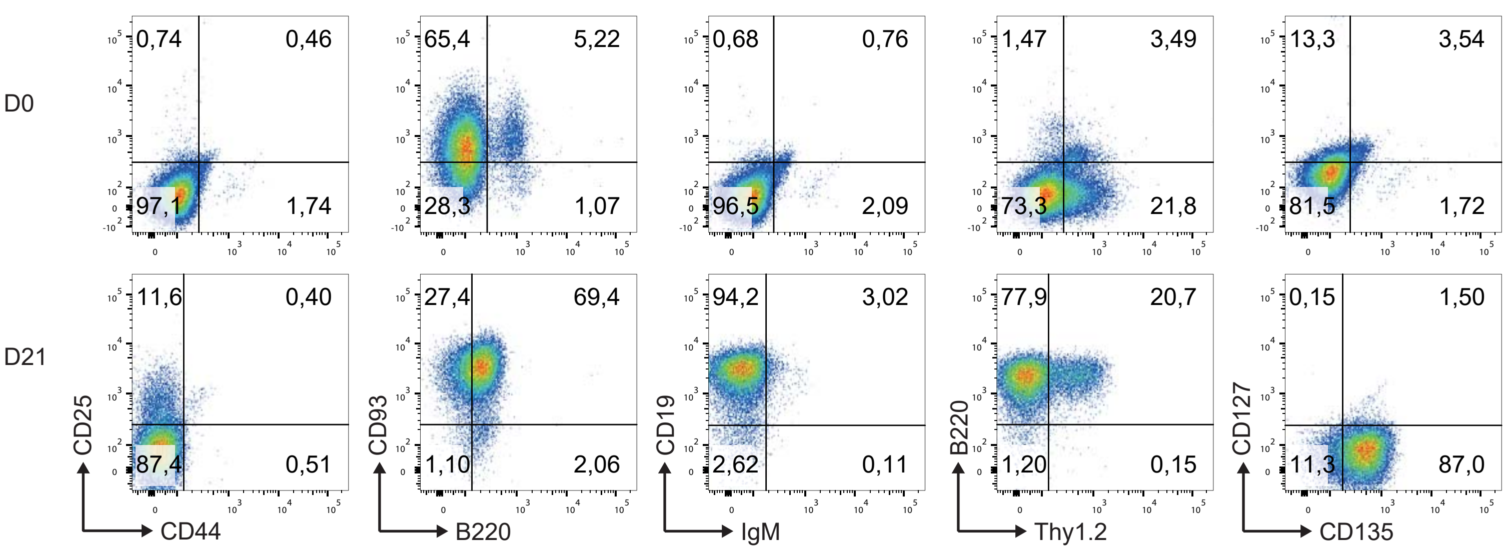


**C**

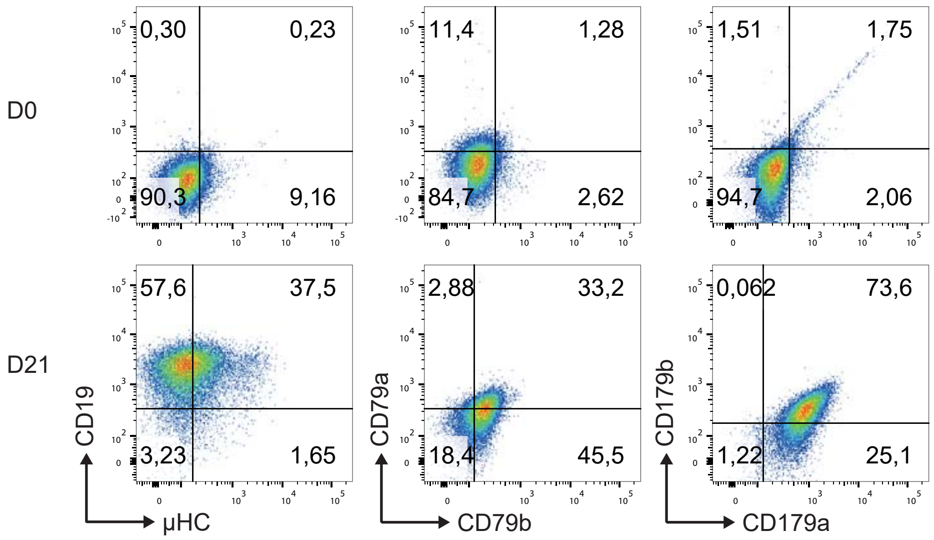


Figure S6: Differentiation was induced in FL-P cells (wt) for 21 days in the presence of IL-7, FLT3L and SCF. Cells were stained for surface (A, B) or intracellular markers (C). All the time points were tested for all staining combinations; shown is a selection.

**Figure S7**

**A**

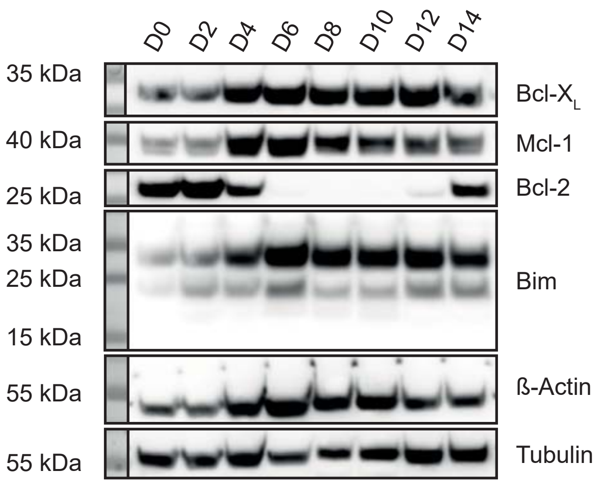


**B**

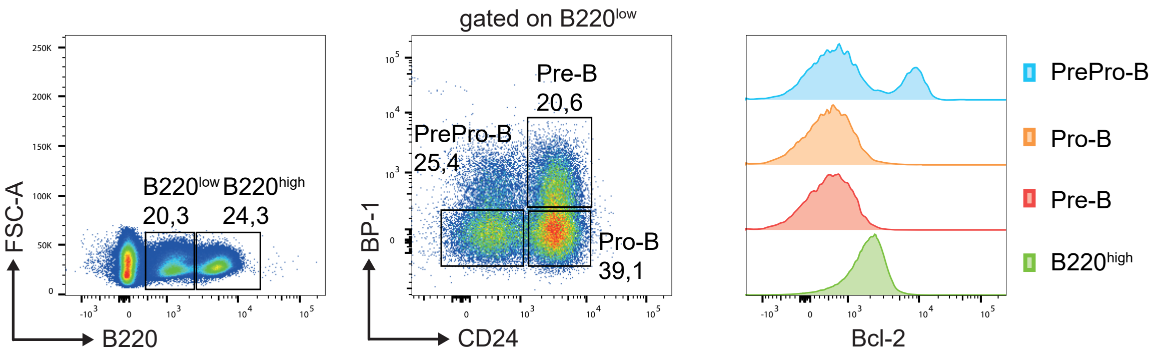


Figure S7: A, Western blot analysis of endogenous Bcl-2 protein levels in Noxa-deficient Hoxb8-FLT3 cells differentiating *in vitro*. FL-D cells were cultured in the presence of IL-7, FLT3L and SCF for 14 days. Samples were taken every two days, lysed and analyzed by SDS-PAGE and Western blotting. Membranes were probed for Bcl-2 family proteins as indicated.

B, Primary bone marrow cells were isolated from a wt C57Bl/6 mouse and stained for surface markers and for Bcl-2-expression. Left panel, Bcl-2-high cells are mostly mature B cells while B200-low cells comprise the developing B cell populations shown in the middle panel. Right panel shows the levels of intracellular Bcl-2 within the B220-high population (directly gated from left panel) and of the immature populations identified in the middle panel (top to bottom, increasing maturity). The results are representative of three independent experiments.

**Figure S8**

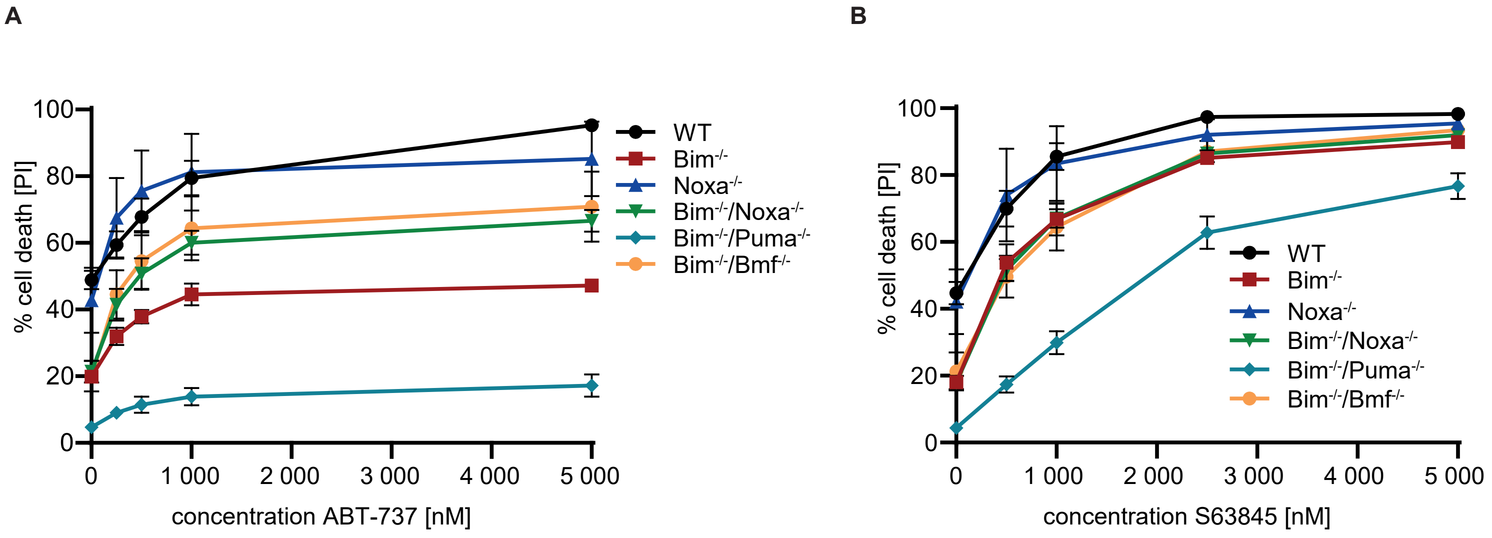


Figure S8: FL-D cells ($10^5$ d14 in vitro differentiated cells per well in 24-well plates) of the indicated genotypes were treated with various concentrations of ABT-737 (A) or S63845 (B). Cell viability was determined after 24h by propidium iodide staining and flow cytometry. Data are means/SEM of three (A) or four to six (B) independent experiments.
